# Supplementary material for: Cd, Cu, and Zn Accumulations Caused by Long-Term Fertilization in Greenhouse Soils and Their Potential Risk Assessment
Source: Int J Environ Res Public Health. 2019 Aug 6;16(15):2805. doi: 10.3390/ijerph16152805 (PMC6695759; doi:10.3390/ijerph16152805)
Supplement: Supplementary file 1 [file ijerph-16-02805-s001.pdf]

Cd, Cu, and Zn Accumulations Caused by Long-term Fertilization in Greenhouse Soils and their Potential Risk Assessment

Zhongbin Liao [1], Yali Chen [1,*], Jie Ma [1], Md. Shafiqul Islam [1], Liping Weng [1] and Yongtao Li [1,2]

[1] Agro-Environmental Protection Institute/Key Laboratory for Environmental Factors Control of Agro-product Quality Safety, Ministry of Agriculture and Rural Affairs, Tianjin 300191；liaozhongbin93@163.com (Z.L.); majie@caas.cn (J.M.); msislamdebd@yahoo.com (M.S.I.); liping.weng@wur.nl (L.W)

[2] College of Natural Resources and Environment , South China Agricultural University, Guangzhou 510642; yongtao@scau.edu.cn (Y.L.)

* Correspondence: chenyali@caas.cn

List of contents

S1. Basic information of sampling sites of this study.

S2. Total variance explained for the heavy metal contents based on PCA analysis.

S3. Annual heavy metal input to greenhouse soils in Shouguang.

S1. Basic information of sampling sites of this study.

Table S1. Basic information of sampling sites of this study

Sampling site	Crop	Fertilizer	Pesticides	Cultivation age
1	Cabbage, pakchoi	Livestock manure	Imidacloprid, Acetamiprid, Fibrin, Glyphosate, Leaf Mould Pasteurella, Chlorpyrifos, Diphenylether Metricyclic Files	29 years
2	Soybean	Livestock manure	-	30 years
3	Soybean	Livestock manure, N-P-K compound fertilizers, Trace element compound fertilizers	-	Over 30 years
4	Cucumber	Livestock manure	Dimethomorph	2 years
5	Color pepper	Livestock manure	Dimethomorph, Dichlorvos	3 years
6	Cucumber	Livestock manure	Clotrile, Avermectin, Quinolone	11 years
7	Pepper	Livestock manure, N-P-K compound fertilizers, Bacterial manure	Acaricide	10 years
8	Cucumber	Livestock manure, N-P-K compound fertilizers	-	5 years
9	Cucumber	Livestock manure, Bacterial manure	-	10 years
10	Bell pepper	Livestock manure	-	3 years
11	Tomatoes	Livestock manure	-	30 years
12	Cucumber, Towel gourd	Livestock manure	Chlorothalonil, Simulin	8 years
13	Cucumber, Towel gourd	Livestock manure, Rooting Fertilizer	-	10 years
14	Pepper	Livestock manure	-	13years
15	Cherry tomatoes	Livestock manure	-	13 years
16	Cucumber, Towel gourd	Livestock manure	Dichlorvos, Iprodione	1 years
17	Cucumber	Livestock manure	-	1 years
18	Towel gourd	Livestock manure	-	4 years
19	Cucumber	Livestock manure	-	3 years
20	Cucumber,	Livestock manure, N-P-K	-	4 years

	Balsam pear	compound fertilizers		
21	Eggplant	Livestock manure	-	15 years
22	Eggplant	Livestock manure	-	10 years
23	Pepper	N-P-K compound fertilizers	-	18 years
24	Gourd, Persimmon	Livestock manure, N-P-K compound fertilizers	Imidacloprid, Fluoromycin	13 years
25	Gourd, Persimmon	Livestock manure	Powdery Mildew Drug	15 years
26	Snakegourd	Livestock manure	-	5 years
27	Gourd,	Livestock manure, Rice husk		4.5 years
28	Tomatoes	Livestock manure	-	17 years
29	Tomatoes	Livestock manure	-	10 years
30	Cherry tomatoes	Livestock manure	-	3 years

- not use or no data

S2. Total variance explained for heavy metal contents based on PCA analysis.

Table S2. Total variance explained for heavy metal contents based on PCA analysis.

Component	Initial Eigenvalues			Extraction Sums of Squared Loadings			Rotation Sums of Squared Loadings		
	Total	% of variance	Cumulative %	Total	% of variance	Cumulative %	Total	% of variance	Cumulative %
**1**	4.517	45.170	45.170	4.517	45.170	45.170	4.516	45.163	45.163
**2**	2.398	23.976	69.147	2.398	23.976	69.147	2.398	23.984	69.147
**3**	0.977	9.775	78.921						
**4**	0.794	7.936	86.858						
**5**	0.506	5.057	91.915						
**6**	0.370	3.701	95.616						
**7**	0.199	1.994	97.610						
**8**	0.131	1.309	98.919						
**9**	0.064	0.642	99.561						
**10**	0.044	0.439	100.000						

S3. Annual heavy metal input to greenhouse soils in Shouguang.

Table S3. Annual heavy metal input to greenhouse soils in Shouguang.

	Source	Cd	Cu	Zn
**Concentration ($mg\cdot kg^{-1}$)[a]**	Compound fertilizers	0.24	10.7	207
	Livestock manures	1.06	315.6	685.4
**Input intensity ($mg\cdot m^{-3}$)[b]**	Compound fertilizers	0.051	2.27	43.88
	Livestock manures	4.393	1307	2840
**Increment ($mg\cdot kg^{-1}\cdot yr^{-1}$)[c]**		0.004	1.14	2.51
**Soil concentration ($mg\cdot kg^{-1}$)**		0.21	42.1	144.4
**Limit concentration[d]**		0.3	100	250
**Time required (yr)[e]**		23	51	42

[a]Concentrations of compound fertilizers and livestock manures from [1].

[b]Calculated with the total input reported in [2].

[c]Calculated with a soil density of 1.15 $g\cdot cm^{-3}$ and soil depth of 20 cm.

[d]The concentration limit defined in the Environmental Quality Evaluation Standard for Farmland of Greenhouse Vegetables Production (HJ333-2006).

[e]Time required to increase the soil heavy metal concentrations from their current levels (Table 2) to the concentration limit defined in HJ333-2006.

References

1. Peng, H.; Chen, Y.; Weng, L.; Ma, J.; Ma, Y.; Li, Y.; Islam, M. S., Comparisons of heavy metal input inventory in agricultural soils in North and South China: A review. *Science of the Total Environment* **2019,** 660, 776-786.
2. Zeng, X. B.; Bai, L. Y.; Li, L. F.; Su, S. M., The status and changes of organic matter, nitrogen, phosphorus and potassium under different soil using styles of Shouguang of Shangdong Province. *ACTA ECOLOGICA SINICA* **2009,** 29, (7), 3737-3746. (in Chinese).
